# Cooperative antibiotic resistance in bacteria: beyond biofilms

**DOI:** 10.3389/fmicb.2026.1906606

**Published:** 2026-08-11

**Authors:** Qinqin Wang, Valentine Cyriaque, Jonas Stenløkke Madsen

**Affiliations:** 1 Section of Microbiology, Department of Biology, University of Copenhagen, Copenhagen, Denmark; 2 Centre Eau Terre Environnement, Institut National de le Recherche Scientifique, Québec City, QC, Canada; 3 Proteomics and Microbiology Laboratory, Research Institute for Biosciences, UMONS, Mons, Belgium

**Keywords:** antibiotic resistance, extracellular, horizontal gene transfer (HGT), microbial cooperation, public goods

## Abstract

Cooperative behaviors among microorganisms, such as biofilm formation, are widespread and play a key role in the emergence and evolution of antibiotic resistance by promoting genetic exchange and environmental adaptability. Bacterial cooperation can involve the secretion of metabolically costly “public goods” that benefit neighboring cells. These include *β*-lactamases, chloramphenicol acetyltransferase, outer membrane vesicles, metabolites, and signaling molecules, which can protect susceptible bacteria by reducing local antibiotic concentrations and thereby attenuating selection pressure. Importantly, genes encoding these extracellular products are often subject to horizontal gene transfer, facilitating cooperative interactions across species within microbial communities. In this review, we summarize current knowledge of these extracellular products, highlight their roles in the development and evolution of cooperative antibiotic resistance, examine their potential implications for antibiotic therapy, and identify key gaps in current research. We also examine future research directions and consider how integrating microbial social interactions and community dynamics in antimicrobial strategies could offer new ways to reduce the emergence and spread of antibiotic resistance.

## 1 Introduction

Antibiotic resistance is a growing global threat under the One Health framework, exacerbated by the slow development of new antibiotics (Aslam et al., 2021; Murray et al., 2022). This has created an urgent need for alternative strategies to preserve the efficacy of existing drugs, including approaches that delay or mitigate resistance evolution. Antibiotic resistance has long been regarded as a genetically encoded, cell-autonomous trait. However, recent studies highlight the importance of cooperative antibiotic resistance (CoopAR) (Vega and Gore, 2014; Nicoloff and Andersson, 2016; Denk-Lobnig and Wood, 2025), a community-level phenomenon in which antibiotic-resistant bacteria produce factors that function directly or indirectly as public goods by modifying the local environment or the physiology of neighboring cells, reducing the effective impact of antibiotics, thereby enabling susceptible or less-resistant cells to remain active or grow under otherwise inhibitory antibiotic concentrations (Bottery et al., 2021). Both experimental and theoretical work has demonstrated that resistant bacteria can protect susceptible neighbors under antibiotic stress, thereby facilitating collective survival dynamics through mechanisms of antibiotic tolerance and persistence (Box 1) (Huemer et al., 2020; Wang et al., 2023, 2025; Zhao et al., 2023). Importantly, CoopAR also introduces distinct ecological and evolutionary dynamics, including cross-species cooperation, exploitation by non-producing “cheaters,” and the emergence of phenotypic heterogeneity. In this context, persister cells further link transient tolerance to the evolution of long-term resistance (Niu et al., 2024).

Box 1 **Antibiotic resistance/tolerance/persistence**

Bacteria employ various strategies to survive antibiotic treatment (Brauner et al., 2016). They either (i) actively counteract their toxic effect (*aka.* resistance), (ii) survive transient exposure to highly concentrated antibiotics, usually by slowing down their growth or elongating the lag phase (*aka.* tolerance) or (iii) heterogeneously respond to the antibiotic toxicity, allowing a subpopulation of cells to persist in the presence of antibiotics (*aka.* persistence). Resistance allows bacteria to grow at higher antibiotic concentrations, using inherited efflux pumps or degradative enzymes such as *β*-lactamase. Therefore, resistance is typically quantified by minimal inhibitory concentration (MIC). By contrast, tolerance does not affect the MIC but instead prolongs the time required to kill a bacterial population. Tolerance can arise from inherited or non-inherited mechanisms. Persistence, however, is not inherited. When persister cells regrow and are re-exposed to the same antibiotic conditions, the population again exhibits a heterogeneous response. This is because persistence is associated with transient physiological states (e.g., dormancy or slow growth) rather than stable genetic changes.

Other cooperative phenotypes among bacteria can directly enhance CoopAR and the emergence of public antibiotic resistance traits, like biofilm formation are among the most well-known (Box 2) (Madsen et al., 2018). Within biofilms, the extracellular matrix, spatial organization, and reduced diffusion collectively enhance the effectiveness of shared resistance determinants and facilitate cooperative protection against antibiotics (Liu et al., 2024). However, these structural features are not prerequisites for CoopAR. Importantly, many cooperative resistance mechanisms, including extracellular antibiotic degradation, metabolite sharing, quorum sensing, and outer membrane vesicle-mediated delivery of resistance factors, also operate in planktonic populations and polymicrobial communities, where cooperation is independent of biofilm formation. Thus, while biofilms provide a well-established ecological framework in which cooperative resistance is readily observed, CoopAR extends beyond biofilm-associated lifestyles.

Box 2 **Biofilms - antibiotic tolerance**

Biofilms are adherent communities of bacterial cells encased in a self-produced extracellular polymeric substance (EPS), which can be considered a public-good. Importantly, biofilm-associated protection and cooperative antibiotic resistance (CoopAR) are overlapping but not synonymous concepts. The EPS matrix itself is a biofilm-specific public good that can directly reduce antibiotic efficacy by limiting antibiotic diffusion, altering local chemical conditions, and creating spatially heterogeneous microenvironments. In contrast, many CoopAR mechanisms discussed in this review, including extracellular *β*-lactamases, antibiotic-modifying enzymes, extracellular vesicles, and diffusible metabolites, can operate both within and outside biofilms. Biofilms further facilitate CoopAR by promoting high cell densities, spatial structure, and retention of public goods, thereby enhancing cooperative interactions and community-level protection.

The formation and development of biofilms include bacterial attachment, maturation, and dispersion (Liu et al., 2024). Biofilms can release and disperse bacteria into the surrounding environment, allowing spread to new locations and formation of new communities, thus facilitating the dissemination of pathogenic bacteria and infections. Therefore, biofilms can act as reservoirs of bacteria, enabling the re-release of planktonic cells and contributing to persistent or recurrent infections. On the other hand, the viscoelastic biofilm matrix serves as a diffusion barrier that limits antibiotic penetration and contributes to bacterial tolerance and immune evasion (Gunn et al., 2016). This directly makes the treatment of biofilm-associated infections more difficult.

The viscoelastic properties of biofilms contribute to bacterial antibiotic tolerance, in combination with several other factors as outlined below. (i) EPS leads to increased viscoelasticity in biofilms, and the formation of dense bacterial groups strongly delays the internal infiltration of antibiotics, thus reducing the concentration of antibiotic inside the biofilms. (ii) Steep gradients of, for example, oxygen and nutrients in biofilms can reduce the rate of bacterial metabolism and limit the bactericidal efficacy of the antibiotics. (iii) Physiological and taxonomic diversity within biofilms can activate stress responses and resistance-associated pathways that increase tolerance to environmental stressors, including antibiotics. (iv) Tight cell-to-cell proximity promotes horizontal gene transfer, while extracellular DNA within the EPS matrix can serve as a substrate for genetic exchange.

**Figure 1:**
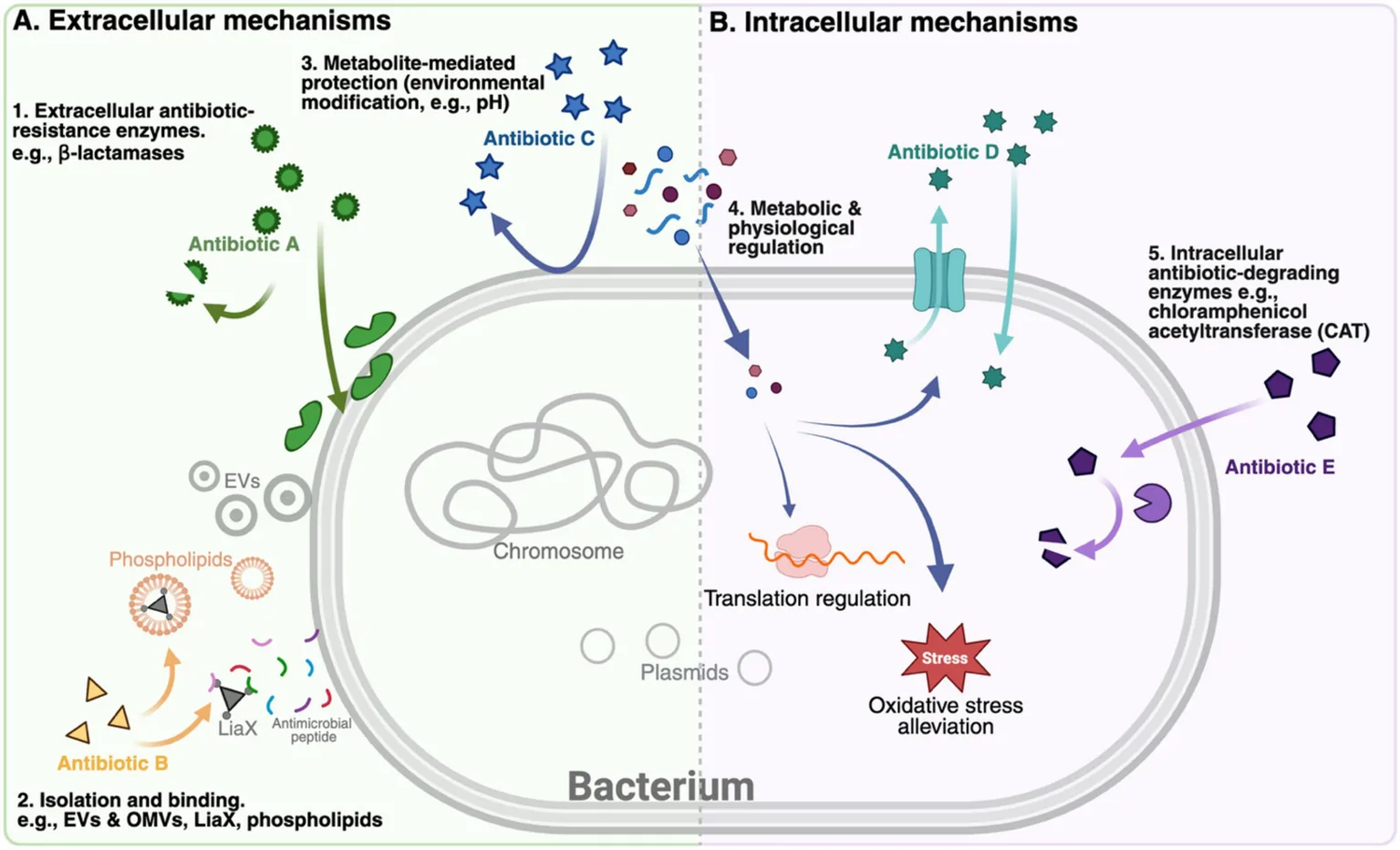
Mechanisms of cooperative antibiotic resistance (CoopAR) mediated by public goods. Public goods produced by resistant bacteria reduce local antibiotic efficacy, thereby protecting neighboring susceptible bacteria and promoting CoopAR. These mechanisms can be broadly classified into **(A)** extracellular and **(B)** intracellular mechanisms. **(A)** Extracellular mechanisms. (1) Extracellular enzymatic degradation of antibiotics by secreted enzymes, such as *β*-lactamases that hydrolyze *β*-lactam antibiotics. (2) Extracellular sequestration of antibiotics by membrane phospholipids or extracellular vesicles (EVs), reducing antibiotic availability, as reported for daptomycin. (3) Metabolite-mediated environmental modification, e.g., extracellular pH changes, which reduce antibiotic uptake or activity, such as tetracycline. **(B)** Intracellular mechanisms. (4) Metabolite-mediated physiological regulation, in which diffusible metabolites modulate gene expression, activate multidrug efflux pumps, and alleviate oxidative stress, thereby reducing intracellular antibiotic accumulation and enhancing bacterial survival. Representative substrates include erythromycin, ampicillin, and ciprofloxacin. (5) Intracellular antibiotic modification by antibiotic-modifying enzymes, such as chloramphenicol acetyltransferase, which inactivates chloramphenicol intracellularly and lowers extracellular antibiotic concentrations, thereby decreasing environmental antibiotic concentrations and promoting community-level protection.

A number of excellent reviews have comprehensively summarized antibiotic resistance associated with biofilms (Liu et al., 2024; Almatroudi, 2025; Bhuiyan, 2025; Elshobary et al., 2025; Nithyanand et al., 2025). Therefore, rather than revisiting these mechanisms in detail, this review focuses on cooperative antibiotic resistance mechanisms that are independent of biofilm formation. Specifically, we discuss how publicly shared resistance factors, including antibiotic-degrading enzymes, extracellular factors, metabolites, and signaling molecules, sometimes conveyed by outer membrane vesicles (Figure 1). These factors function as public goods by reducing local antibiotic efficacy and creating protective microenvironments, thereby generating collective benefits that neighboring cells can exploit and promoting bacterial cooperative behavior (Smith and Schuster, 2019). In addition, cooperative metabolic interactions between microbial species can promote survival under antibiotic exposure by altering cellular physiology and stress responses (Perry et al., 2022).

CoopAR is often facilitated by mobile genetic elements, and this way the resistance traits are disseminated across species boundaries (Lee et al., 2021, Zhu et al., 2024). Notably, CoopAR can itself promote horizontal gene transfer (HGT), as susceptible cells rescued by cooperative resistance have been shown to become more likely to acquire CoopAR encoding conjugative plasmids (Wang et al., 2023). Beyond molecular mechanisms, CoopAR is also influenced by eco-evolutionary dynamics. Environmental fluctuations, demographic noise, and spatial structure can destabilize cooperative resistance and even promote the extinction of resistant populations under certain conditions (Hernández-Navarro et al., 2023, 2026). More generally, microbial interactions, including both cooperation and competition, are critical in modulating antibiotic activity and resistance evolution (Zhang et al., 2026).

Leveraging the social traits of CoopAR may offer novel therapeutic opportunities to disrupt cooperative resistance (Ma et al., 2024; Griffin and Leeks, 2025). For example, *β*-lactamases can benefit microbial communities by providing protection during *in vivo* antibiotic treatment (Cubillos-Ruiz et al., 2022). However, these mechanisms may also present significant risks in polymicrobial infections by facilitating the persistence and spread of antibiotic resistance. Additionally, population-level resistance dynamics, such as inoculum effects and spatial organization, further impact treatment efficacy (Geyrhofer et al., 2023; Brepoels et al., 2025; Denk-Lobnig and Wood, 2025). Given these complexities, integrating eco-evolutionary principles into therapy design has been proposed as a promising strategy to combat resistance (Sanz-García et al., 2023) as discussed further in this review.

Although CoopAR has gained increasing attention, current studies remain fragmented across mechanistic, ecological, and evolutionary levels. This fragmentation is particularly concerning given the widespread resistance to *β*-lactams, much of which is mediated by cooperative resistance mechanisms such as *β*-lactamases. Because *β*-lactams remain among the most clinically significant classes of antibiotics, CoopAR represents a major and underappreciated global health challenge. Moreover, an increasing diversity of CoopAR mechanisms is being discovered, including antibiotic-modifying and -degrading enzymes (e.g., chloramphenicol acetyltransferases, macrolide esterases, and Tet(X2)), antibiotic sequestration and neutralization by extracellular factors (e.g., phospholipids and LiaX), extracellular vesicle-mediated protection, metabolic and environmental modification, and signaling-mediated resistance (Figure 1). To provide an overview of these diverse mechanisms, the major public goods mediating CoopAR are summarized in Table 1. While analogous cooperative mechanisms have also been described in fungi (Yu et al., 2022; Nithyanand et al., 2025), this review focuses specifically on bacterial CoopAR and the public goods that mediate it. Currently, much research is gene-centric, relying primarily on DNA sequencing, and does not adequately consider the impact of cooperative interactions and community-level dynamics. Here, we examine current knowledge of CoopAR to better understand the nature and function of the relevant public goods, their emergence and transmission among bacteria, and how this knowledge may inform strategies for rationally combating cooperative antibiotic resistance.

**Table 1:** Public goods mediating cooperative antibiotic resistance (CoopAR).

CoopAR mechanism	Public goods	Representative bacteria	Antibiotic(s)	Clinical relevance	References
Extracellular antibiotic degradation	*β*-lactamases; alkaline protease Apr	*Escherichia coli*, *Bacillus licheniformis*	*β*-lactams; polymyxins	Strong evidence; major contributor to polymicrobial infections and *β*-lactam treatment failure	Yurtsev et al. (2013); Yin et al., (2019); Gjonbalaj et al., (2020); Wang et al. (2025)
Intracellular antibiotic inactivation	Chloramphenicol acetyltransferase (CAT); Tet(X2); Ere(A)	*Streptococcus pneumoniae*, *Escherichia coli*	Chloramphenicol; tetracyclines; macrolides	Moderate evidence; depends on antibiotic permeability and degradation efficiency	Sorg et al. (2016); Nicoloff and Andersson, (2016); Blake et al. (2024)
Antibiotic sequestration and neutralization	Phospholipids; stress-response proteins; LiaX; extracellular vesicles (EVs)	*Staphylococcus aureus*, *Enterococcus faecalis* *Acinetobacter baumannii*	Daptomycin; antimicrobial peptides	Contributes to protection in biofilms and polymicrobial communities	Pader et al. (2016); Khan et al. (2019); Schwechheimer and Kuehn, (2015); Kim et al. (2018); Baltz, (2009)
Environmental and physiological modification	Trimethylamine; ammonia; 2′ amino-acetophenone (2-AA); indole; volatile organic compounds; H₂S	*Pseudomonas aeruginosa*, *Escherichia coli*, *Bacillus subtilis*, *Burkholderia ambifaria*	*β*-lactams; tetracyclines; aminoglycosides	Emerging evidence; promotes long-range community protection and stress adaptation	Bernier et al. (2011); Perry et al. (2022); Weisskopf et al. (2021)

## 2 Social dynamics of cooperative antibiotic resistance

**Figure 2:**
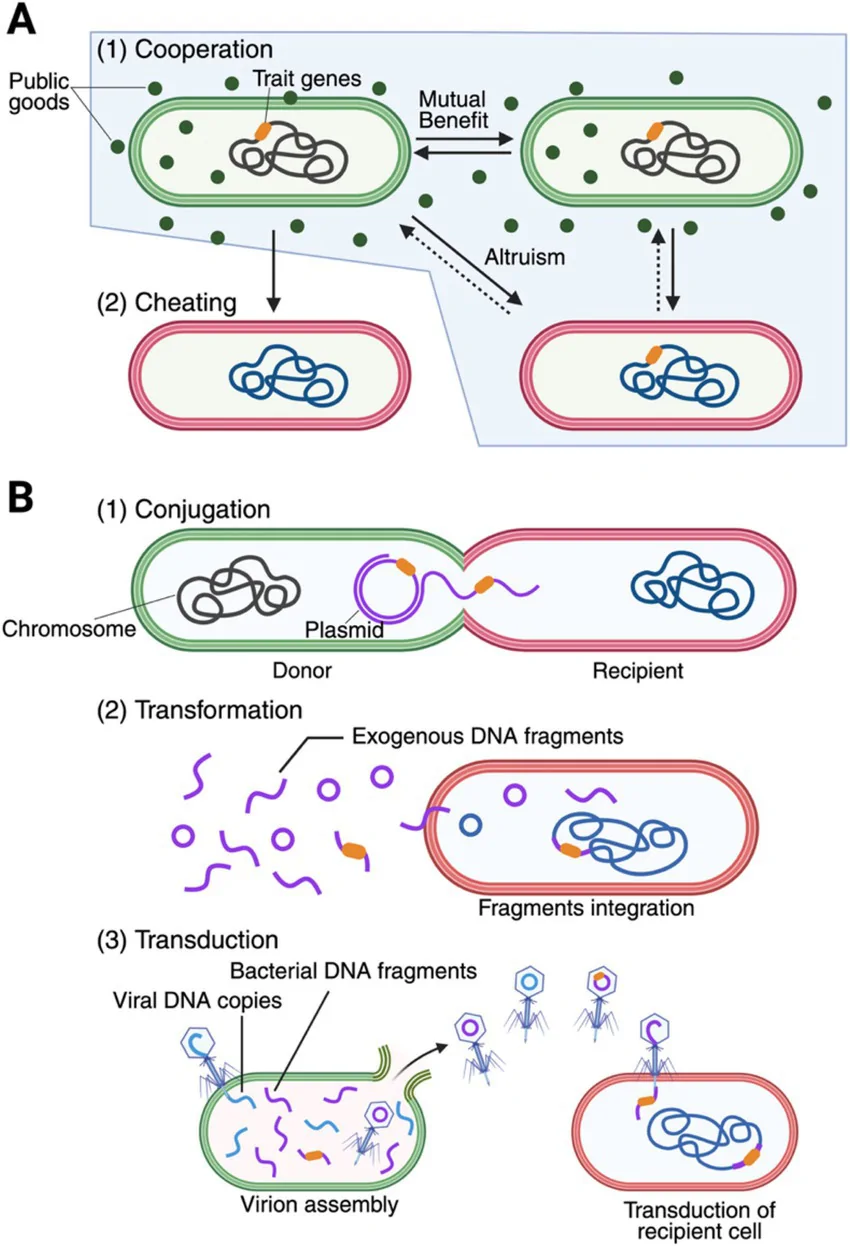
Social dynamics in cooperative antibiotic resistance (CoopAR). **(A)** Cooperation and cheating. Cooperation can be divided into mutual benefit among producers and altruistic behavior between producers and non-producers. Altruism occurs mainly between producers and non-producers carrying trait genes and can exist across species. These cooperative non-producers will benefit the inclusive fitness of producers. Since public goods often work extracellularly, cheaters who do not carry trait genes take advantage of public goods and do not benefit the producers. Solid green circles represent public goods. Solid arrows are direct benefits, and dashed arrows are indirect benefits. **(B)** Horizontal gene transfer: (1) Plasmid-mediated conjugation; (2) Transformation; (3) Transduction. Horizontal transfer of antibiotic-resistant genes can promote bacterial cross-species cooperation and convert susceptible bacteria to resistant bacteria. **(A, B)** Green bacteria are public goods producers. Red bacteria refer to other bacteria, including the same species and different species. Orange marks are trait genes, mainly referring to antibiotic resistance genes.

**Figure 3:**
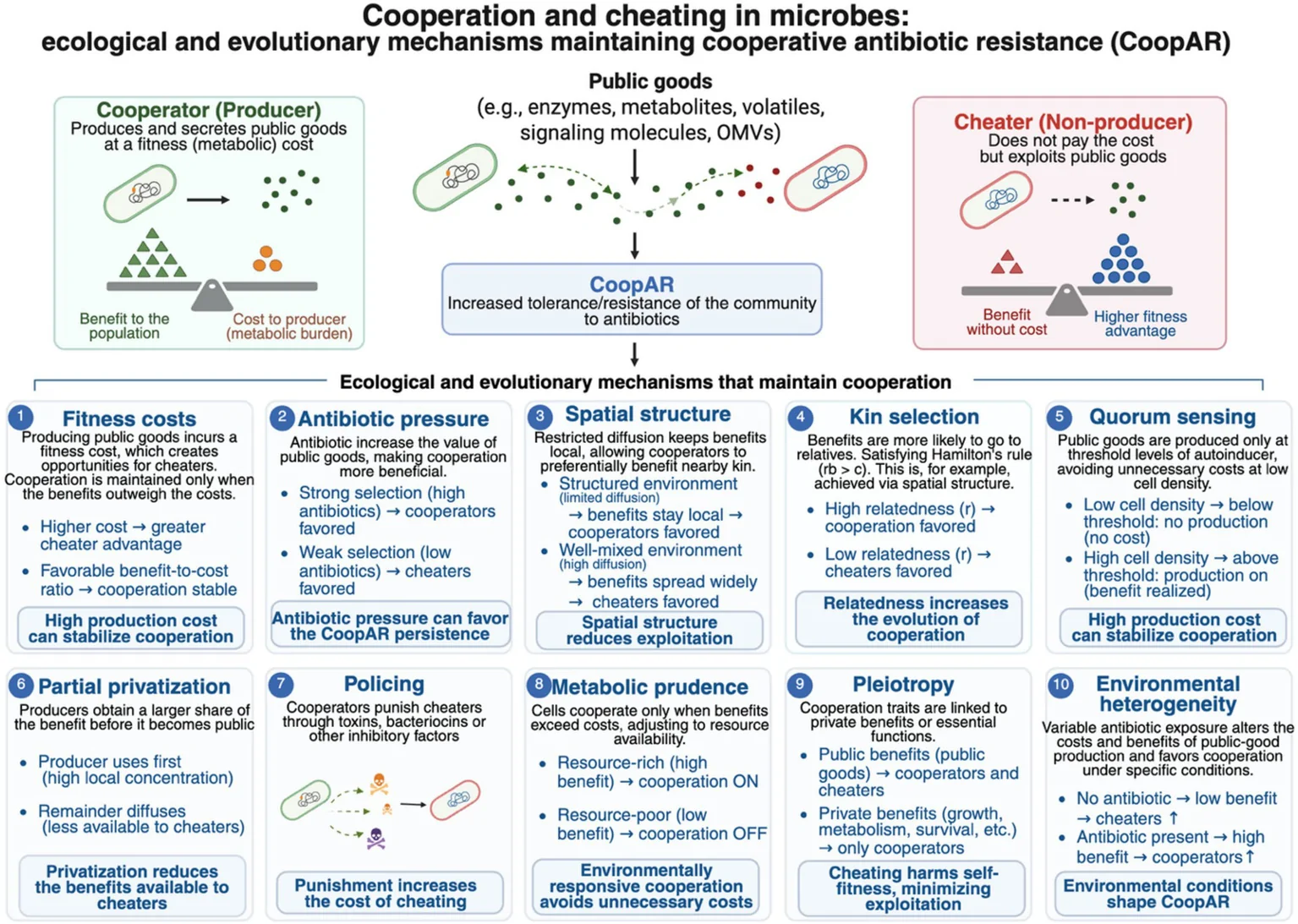
Schematic illustration of the ecological and evolutionary mechanisms maintaining cooperative antibiotic resistance (CoopAR). The ecological and evolutionary mechanisms that maintain CoopAR include fitness costs, antibiotic selection pressure, spatial structure, kin selection, quorum sensing, partial privatization, policing, metabolic prudence, pleiotropy, and environmental heterogeneity.

Antibiotic-sensitive bacteria may persist near cells expressing CoopAR factors (Madsen et al., 2018; Windels et al., 2019). From an evolutionary perspective, the maintenance of such cooperative protection can be explained by Hamilton’s theory of cooperation, which proposes that cooperation evolves through either direct fitness benefits to the producer or indirect fitness benefits arising from increased inclusive fitness among genetically related individuals (Hamilton, 1964). Accordingly, CoopAR may confer fitness advantages on both resistant producers and genetically related neighboring cells (Figure 2A). When cooperative resistance determinants are horizontally transferred, these benefits may also extend to neighboring cells that carry the same cooperative resistance determinants (Figure 2B). The persistence of CoopAR depends on a complex interplay of ecological and evolutionary processes (Figure 3).

However, because public goods are accessible to nearby cells regardless of their contribution, cooperative resistance is vulnerable to exploitation by non-producing “cheaters,” including members of other species (Perlin et al., 2009) (Figure 2A; Box 3). From an evolutionary standpoint, it is sensible to share a costly public good with kin, however, helping non-kin unconditionally is unfavorable.

Box 3 **Public goods and the tragedy of commons**

Bacteria live in dense communities where diverse social interactions occur, including cooperative virulence (Bakkeren et al., 2022), nutrient acquisition, and detoxification processes (Alexandrino et al., 2014; Domingues et al., 2017). The products of such cooperative behaviors are referred to as public goods. Originally from economics, the term describes resources that are costly to produce but freely accessible to all members of a community (Smith and Schuster, 2019). In microbiology, public goods are metabolically expensive molecules or activities that benefit neighboring cells, such as exopolysaccharides, surfactants, toxins, anti-toxins, nutrients, and degradative enzymes. These public goods may be tightly regulated by quorum sensing and can act as facilitators for either conspecific or heterospecific cells, with varying degrees of privatization.

The tragedy of commons is the exploitation of public goods by non-cooperators (cheaters), ultimately leading to the collapse of cooperation due to the fitness advantage of cheating. Cheaters arise from cooperative populations after deletion or inactivation of one of the genes responsible for the production/regulation of public goods. Mutants then still benefit from the cooperative behavior of their neighbors with no fitness cost. But, as cheaters increase in number, the benefit provided by cooperators decreases per bacterial cell until it gets too low to be effective for the population. Several mechanisms have been proposed that could avert the selection of cheaters, such as increasing the fitness of the cooperators (privatization) or promoting the relatedness (propensity to have the same and operative cooperative gene) of neighbors through spatial organization or HGT.

To counteract exploitation by cheaters, kin selection is thought to be an important mechanism for maintaining bacterial cooperation. By increasing the likelihood that benefits are directed toward genetically related individuals, kin selection helps preserve cooperative traits despite the cost of public-good production (Buckling and Brockhurst, 2008; Smith and Schuster, 2019). Because bacteria grow by clonal division and frequently inhabit structured communities, such as biofilms, public goods are more likely to be shared among neighboring kin rather than exploited by cheaters.

In addition, horizontal gene transfer (HGT) of public good genes can convert non-producers into producers. This process may increase local relatedness at the functional level and stabilize cooperation, particularly because many antibiotic-resistance determinants are carried on conjugative plasmids (Lee et al., 2021) (Figure 2B).

Even among isogenic populations where all cells carry the same antibiotic resistance gene, the production of CoopAR can confer population-level advantages. In such populations, the level of resistance can vary among individual cells or subpopulations despite identical genotypes, a phenomenon known as transient antibiotic heteroresistance (Nicoloff et al., 2024). This has been well documented for CoopAR-associated traits such as *β*-lactamase production (Roch et al., 2023; Choby et al., 2025). It is plausible that variation in expression levels could promote a form of division of labor. Cells producing high levels of cooperative resistance factors may protect neighboring cells with lower expression, who in turn avoid the metabolic costs of production and can instead allocate resources to growth or alternative phenotypes (Nicoloff et al., 2024). Such interactions could enhance overall population fitness under antibiotic stress.

## 3 Mechanisms of cooperative antibiotic resistance

### 3.1 CoopAR by extracellular degradation of antibiotics

Extracellular enzymatic degradation of antibiotics is one of the most important mechanisms underlying CoopAR, with *β*-lactamases being the most prevalent and clinically significant antibiotic-resistance enzymes. *β*-lactamases hydrolyze the *β*-lactam ring, thereby inactivating *β*-lactam antibiotics, which remain the most widely used class of antibacterial agents (Bush and Bradford, 2016; Bush, 2018; Tooke et al., 2019).

*β*-lactamase producers can protect susceptible bacteria across species, increasing the failure rate of *β*-lactam antibiotic treatment for microbial infections (Brook, 2004; Perlin et al., 2009; Medaney et al., 2016). Although *β*-lactamases primarily function in the periplasm, it can also be translocated outside the cell (Livermore, 1995). Extracellular β-lactamase can inactivate antibiotics in the surrounding environment, thereby protecting nearby susceptible bacteria (Yurtsev et al., 2013). Importantly, different *β*-lactamases can provide cross-protection against a range of *β*-lactam substrates, further broadening community-level CoopAR. At the same time, *β*-lactamases vary substantially in their ability to hydrolyze *β*-lactams extracellularly (Wang et al., 2025).

In addition to free extracellular *β*-lactamases, some were found to be packaged into extracellular vehicles (EVs) that are released into the environment where they can inactivate extracellular antibiotics. Several studies have proved that EVs released by *β*-lactam resistant bacteria carry active *β*-lactamases to hydrolyze antibiotics in the environment, which protects bacteria that cannot produce β-lactamase from lethal doses of antibiotics (Schaar et al., 2011, 2014; Stentz et al., 2015). Unlike most *β*-lactamases, NDM-1 is membrane-anchored via lipidation, which further promotes its incorporation into EVs and facilitates the extracellular dissemination of carbapenemase activity (González et al., 2016; Prunotto et al., 2020).

The ecological consequences of extracellular *β*-lactamase production have been demonstrated in diverse environments, including liquid broth media (Perlin et al., 2009; Yurtsev et al., 2013; Domingues et al., 2017), solid agar medium (Medaney et al., 2016), and biofilms (Amanatidou et al., 2019). In these settings, *β*-lactamase-producing bacteria produce public goods that enable susceptible “cheater” cells to survive antibiotic exposure, thereby enhancing the overall fitness and resilience of microbial populations and communities (Brook, 2004; Frost et al., 2018).

Other than *β*-lactamases, it has also been reported that polymyxin resistance, broadly distributed among gram-positive bacteria may be caused by the hydrolytic inactivation of antibiotics by the alkaline protease Apr, which can be secreted extracellularly (Yin et al., 2019), hereby functions as CoopAR.

### 3.2 CoopAR by intracellular inactivation of antibiotics

While *β*-lactamases are best known, in relation to CoopAR, for mediating extracellular antibiotic degradation, their intracellular inactivation is also an important facilitator of CoopAR (Wang et al., 2025). More broadly, several intracellular antibiotic-modifying or degrading enzymes can produce similar cooperative effects by lowering the concentration of active antibiotics in the environment. One of the best characterized examples is chloramphenicol acetyltransferase (CATs), which confers resistance by acetylating and inactivating chloramphenicol in the cytoplasm (Murray and Shaw, 1997). Chloramphenicol is a broad-spectrum antibiotic that inhibits protein synthesis by reversibly binding to the 50S ribosome subunit (Shaw, 1983). Because this interaction is reversible, bacterial growth may resume once the extracellular chloramphenicol concentration falls below inhibitory levels, although recovery is also influenced by intracellular antibiotic levels and the physiological state of the cells (Rahal and Simberkoff, 1979; Moffa and Brook, 2015). Consequently, intracellular inactivation of chloramphenicol by CAT enzymes can lower environmental antibiotic concentrations and locally create opportunities for susceptible cells to survive, recover and grow.

A striking example was reported involving chloramphenicol acetyltransferase CatA1, expressed by chloramphenicol-resistant bacteria, which protected susceptible *Streptococcus pneumoniae* populations by inactivating chloramphenicol intracellularly (Sorg et al., 2016; Yurtsev et al., 2016). *In vivo*, rapidly growing resistant cells initially inactivated large amounts of chloramphenicol and were preferentially targeted by host antimicrobial peptides, whereas susceptible cells remained growth arrested. As environmental chloramphenicol concentrations declined, the susceptible subpopulation ultimately benefited from detoxification by the resistant subpopulation.

In addition to chloramphenicol acetyltransferases, cross-species CoopAR has also been observed in bacteria expressing other antibiotic modification or degradation enzymes, such as tetracycline-inactivating enzyme (TET(X2)) or macrolides-inactivating enzyme (Ere(A)) (erythromycin and clarithromycin) (Nicoloff and Andersson, 2016). It was found that the expression of these antibiotic modification or degrading enzymes also reduces the concentration of active antibiotics in the extracellular medium, and the sensitive cells resumed growth when the concentration was lower than their minimum inhibitory concentration (Nicoloff and Andersson, 2016). The recent increase in the number and diversity of reported tetracycline destructase enzymes suggests that additional resistance mechanisms, with potential roles in CoopAR, remain to be characterized (Blake et al., 2024).

Moreover, intracellular mediated CoopAR is based on the high permeability of antibiotics to bacterial cells and the degradation efficiency of intracellular degrading enzymes, among which high permeability is an important factor in determining the entry of antibiotics into cells. The concentration of antibiotics inside the cells determines the amount of antibiotics that enzymes can modify or degrade and thus remove from the environment. For example, aminoglycoside antibiotics (e.g., streptomycin, kanamycin, gentamicin) have difficulty penetrating bacterial cells because they are hydrophilic and positively charged (Magnet and Blanchard, 2005). Low intracellular antibiotic concentration makes it difficult for the intracellular enzymes that degrade fosfomycin or modified aminoglycosides to access their substrates, so it is rare to observe CoopAR under these antibiotic conditions (Nicoloff and Andersson, 2016). In contrast, chloramphenicol as an amphiphilic compound, can diffuse into the lipid bilayer and penetrate bacterial cells with a higher frequency (Magnet and Blanchard, 2005). Therefore, chloramphenicol is rapidly degraded by intracellular degrading enzymes. Thereby, chloramphenicol resistant bacteria can efficiently induce CoopAR, similarly to *β*-lactamases in terms of speed. However, because β-lactamase-mediated inactivation occurs in the extracellular medium and directly benefits sensitive partners, its CoopAR efficiency is higher than that of CatA1, Ere(A), and Tet(X2) (Nicoloff and Andersson, 2016). Additionally, the rate of antibiotic efflux may also influence CoopAR. In the case of erythromycin degradation by Ere(A), reduced efflux increases the occurrence of CoopAR (Nicoloff and Andersson, 2016).

### 3.3 CoopAR by public goods that sequester and neutralize antibiotics

Bacteria can also enable antibiotic resistance by releasing extracellular factors that sequester or neutralize antibiotics. In Gram-positive bacteria, daptomycin resistance is often mediated by the release of membrane phospholipids or stress-response proteins that bind and inactivate the antibiotic (Baltz, 2009). For example, *Staphylococcus aureus* releases phospholipids that act as decoys to sequester daptomycin, while *Enterococcus faecalis* secretes LiaX, which binds antimicrobial peptides and activates the LiaFSR stress response system (Pader et al., 2016; Khan et al., 2019). These extracellular molecules can serve as public goods, benefiting neighboring cells hereby facilitating CoopAR.

Similarly, EVs can absorb or degrade antibiotics, thereby lowering their effective concentration in the environment (Schwechheimer and Kuehn, 2015). EV production is often induced under stress, such as sublethal antibiotic exposure, further enhancing community-level protection. Alternatively, EVs reduce effective antibiotic concentrations in cultures by isolating antibiotics or carrying enzymes that can degrade them (Kim et al., 2018). Together, these mechanisms represent public goods-type strategies that promote CoopAR by sequestering antibiotics extracellularly.

Another clear example of CoopAR is in *Staphylococcus aureus* ΔagrA mutant. The Agr quorum-sensing system is a major regulator of virulence and membrane physiology in *S. aureus*, and loss-of-function *agr* mutants frequently arise during chronic infections and antibiotic treatment. These mutants release “bait” membrane phospholipids that inactivate daptomycin. Notably, the culture supernatant was shown to protect neighboring wild-type cells, exposing the public goods–like nature of this mechanism (Pader et al., 2016).

### 3.4 CoopAR by environmental and physiological changes

Extracellular metabolites and diffusible molecules that do not directly act as antibiotic resistance factors can act as public goods that promote CoopAR. These factors protect neighboring cells by modifying the local environment, reducing antibiotic uptake, activating stress responses, or lowering intracellular antibiotic accumulation (Perry et al., 2022). For example, trimethylamine increases population-level tetracycline resistance by altering environmental pH and lowering antibiotic absorption, whereas ammonia decreases antibiotic permeability, thereby enhancing resistance to tetracycline and ampicillin (Bernier et al., 2011; Létoffé et al., 2014).

In addition to altering the extracellular environment, microbial volatile compounds can also function as long-range signaling molecules that directly modulate the physiology of neighboring cells, thereby promoting CoopAR across spatially separated bacterial populations (Weisskopf et al., 2021). Unlike other diffusible public goods, microbial volatile compounds readily diffuse through both liquid and air environments, enabling CoopAR to extend across spatially separated bacterial populations. For example, 2′ amino-acetophenone (2-AA) produced by *Pseudomonas aeruginosa* promotes antibiotic survival across multispecies communities by regulating cellular translation, whereas indole released by *Escherichia coli* induces efflux pump expression in neighboring bacteria, enabling growth under antibiotic stress (Que et al., 2013; Molina-Santiago et al., 2014). Volatiles organic compounds produced by *Burkholderia ambifaria* and *Bacillus subtilis* have been shown to increase resistance to multiple antibiotic classes in neighboring bacteria, including aminoglycosides, *β*-lactams, and tetracycline (Groenhagen et al., 2013, Kim et al., 2013). Similarly, gasotransmitters such as H_2_S function as broadly protective volatiles by alleviating antibiotic-induced oxidative stress, thereby enhancing bacterial survival to diverse antibiotics (Shatalin et al., 2011). Collectively, these findings demonstrate that volatile compounds are a distinct class of public goods that can extend CoopAR beyond local microenvironments, enabling long-range physiological communication among spatially separated cells and multispecies communities.

## 4 Cooperative antibiotic resistance and horizontal transfer

Horizontal gene transfer (HGT), mediated by plasmid conjugation, bacteriophage mediated transduction, natural transformation and outer membrane vesicles (Von Wintersdorff et al., 2016; Domingues and Nielsen, 2017), has a complex but indisputable association with CoopAR (Von Wintersdorff et al., 2016), and it has even been suggested that HGT itself can be regarded as a type of public good (Lee et al., 2021). The exchange of genetic material across species that shuffles genes needed for survival occurs at a high rate and can exceed those of spontaneous mutations (Charpentier et al., 2012; Lerminiaux and Cameron, 2019), and HGT has greatly influenced the genetic diversity of bacterial populations and can rapidly ensure the survival of bacteria when facing for example antibiotics (Von Wintersdorff et al., 2016).

*Plasmids* are extrachromosomal DNA elements capable of semi-autonomous replication and can mediate CoopAR-gene transfer among populations and communities via conjugation. It is common that CoopAR genes, such as those encoding β-lactamases and chloramphenicol acetyltransferases, are encoded on plasmids (Che et al., 2021; Bale et al., 2023; Castañeda-Barba et al., 2024). Plasmid carriage facilitates several situational advantages: it can increase the copy number of genes because plasmids often exist as multiple copies in the cytoplasm, plasmid-mediated HGTs help genes to shuttle between different cells, whether they are the same species or different species, and they allow rapid gain and loss, conferring dynamic adaptability to the host in fluctuating environments (Dewar et al., 2021; Rodríguez-Beltrán et al., 2021). Beyond disseminating CoopAR genes, conjugative plasmids may also promote the evolutionary stability of cooperation by converting non-producing recipients into producers through HGT. Conversely, CoopAR may also enhance plasmid-mediated HGT by increasing the survival of susceptible recipient cells under antibiotic stress, thereby expanding the pool of potential recipients and creating a positive feedback loop between cooperative resistance and HGT (Wang et al., 2023).

For these reasons, it has been argued that conjugative plasmids are more efficient at fixing genes of horizontal origin compared to transformation and transduction, and sensitive bacteria may be converted into resistant producers that continue to exist in the environment (Sørensen et al., 2005). When the plasmid-borne CoopAR gene transfers to a population, the increased production of the public goods by an increasing number of cooperators can reduce its cost at the individual level, helping its stability at the population level. An increased transcription rate and/or copy number of the plasmid can also boost the protection against antibiotics conferred by CoopAR (Domingues et al., 2017). Furthermore, gene copy number variation at the single cell level, enabled by plasmids and transposons, is an important mechanism underlying transient heteroresistance (Nicoloff et al., 2024).

In line with this, plasmid-mediated HGT has been proposed to stabilize cooperative antibiotic resistance by countering cheating through the conversion of non-producing recipients into producers, thereby reducing the fitness advantage of cheaters (Lee et al., 2021). In this context, plasmid transfer can not only prevent cheaters from taking over the population (Diggle et al., 2007), they also increase local relatedness by transferring to non-cooperative neighbors and therefore promote kin selection to maintain the production of costly public goods (Nogueira et al., 2009; Mc Ginty et al., 2013). Even so, plasmids and CoopAR genes can be lost or mutate, enabling cheaters to arise regardless of plasmid association (Bakkeren et al., 2022). The multicopy nature of plasmids and their retransmission by conjugation, however, may slow the selection for cheating. However, recent comparative genomic analyses challenge the view that plasmids universally stabilize cooperation. Instead, plasmid-mediated HGT appears to facilitate the initial invasion and dissemination of cooperative genes more effectively than their long-term maintenance, which depends on ecological factors, plasmid dynamics, and selection pressures on the host–plasmid association (Dewar et al., 2021).

As mentioned previously, many public goods besides CoopAR are costly extracellular proteins. The biosynthetic cost of secreted proteins can be higher than that of highly expressed proteins (Nogueira et al., 2009). Therefore, although plasmid-mediated HGT can promote cooperation by facilitating the initial invasion of cooperative genes into bacterial populations (Dewar et al., 2021), its contribution to the long-term maintenance of cooperation remains debated. Dimitriu and colleagues argued that plasmids may preferentially stabilize low-cost antibiotic resistance traits rather than energetically costly extracellular cooperation (Dimitriu et al., 2019). Consistent with this view, extracellular proteins are not generally overrepresented on plasmids across the bacterial domain (Dewar et al., 2021). However, in some pathogenic species with a broad host range, such as *Escherichia coli*, *Salmonella enterica*, or *Bacillus anthracis*, extracellular proteins were shown to be associated with MGEs (Nogueira et al., 2009; Dewar et al., 2021).

Thus, the evolutionary consequences of plasmid-mediated HGT appear to be highly context-dependent, varying with plasmid transfer and loss rates, host ecology, population structure, and the ecological characteristics and costs of the shared public goods (Dewar et al., 2021). Many clinically important CoopAR determinants, including *β*-lactamases and chloramphenicol resistance genes, are often plasmid-encoded, highlighting the central role of conjugative plasmids in disseminating CoopAR traits (Doublet et al., 2012; Villa et al., 2012, 2013; Rozwandowicz et al., 2018). As major vehicles for the horizontal transfer of antibiotic resistance genes, plasmids facilitate the evolution and dissemination of antibiotic resistance in bacterial populations (Millan et al., 2016). Overall, the prevalence and persistence of plasmid-borne CoopAR are determined not only by plasmid transfer frequency and the initial density of resistant cells, but also by plasmid-associated fitness costs, population structure, and ecological conditions that influence the balance between cooperation and cheating (Domingues et al., 2017; Bakkeren et al., 2019).

Bacterial extracellular vesicles (EVs) can also contribute to the horizontal transfer of antibiotic resistance (Schwechheimer and Kuehn, 2015; Soler and Forterre, 2020). Many studies subdivide EVs into outer membrane vesicles (OMVs) secreted by gram-negative bacteria and membrane vesicles (MVs) secreted by gram-positive bacteria (Toyofuku et al., 2019). A significant advantage of OMVs is that the cargo molecules contained in the vesicles can be safely transported over long distances without being diluted and degraded (Kolling and Matthews, 1999; Schaar et al., 2013; Alves et al., 2016). *E. coli* OMVs facilitate the transfer and expression of genetic material, including antibiotic resistance genes and virulence genes, into recipient bacteria (Yaron et al., 2000; Bielaszewska et al., 2020). Likewise, OMVs in *Acinetobacter baumannii* can promote the transfer of carbapenem resistance by carrying and transferring antibiotic resistance-encoding DNA and enzymes (Rumbo et al., 2011; Schwechheimer and Kuehn, 2015).

Correspondingly, EVs can not only transfer DNA, but also carry various functional cargoes, for example enzymes, virulence factors, siderophores, surface adherence factors, and nucleic acids, and shuttle them between cells (Lim and Yoon, 2015; Schwechheimer and Kuehn, 2015; Yáñez-Mó et al., 2015; Toyofuku et al., 2019). This powerful transportation function allows EVs to mediate CoopAR.

Interestingly, treatment with *β*-lactam antibiotics also increases the secretion of EVs and their *β*-lactamase content. For example, MVs isolated from methicillin-resistant *Staphylococcus aureus* (MRSA) treated with ampicillin can have up to 22.4 times more *β*-lactamase activity than those cultured without antibiotic pressure (Kim et al., 2020). Imipenem treatment resulted in a significant increase in the secretion of β-lactamase-containing OMVs of *Stenotrophomonas maltophilia* (Devos et al., 2015).

## 5 Translational challenges and clinical implications of cooperative antibiotic resistance

Bacterial infections remain a major global health challenge, and their treatment is increasingly compromised by the spread of antibiotic resistance. The growing prevalence of antibiotic resistance and persistence has made many first-line antibiotics ineffective (Naghavi et al., 2024), while healthcare settings have become important reservoirs for the acquisition and dissemination of antibiotic resistant pathogens (León-Sampedro et al., 2021). Because many bacterial infections are caused by bacteria living in spatially structured polymicrobial communities, cooperative interactions may influence antibiotic efficacy *in vivo* and contribute to persistent infection and treatment failure (Brogden et al., 2005).

Current evidence suggests that CoopAR is more likely to occur in clinical settings with high bacterial densities, prolonged bacterial persistence, and in polymicrobial communities (Cui and Kim, 2024). Chronic airway infections, such as those in cystic fibrosis patients, provide favorable ecological conditions because bacteria remain in close proximity and are repeatedly exposed to prolonged *β*-lactam therapy (Ciofu and Tolker-Nielsen, 2019). Similarly, densely colonized intestinal microbial communities and polymicrobial infections provide ecological conditions with close bacterial proximity and frequent interspecies interactions, which may favor CoopAR (Donaldson et al., 2016; Gjonbalaj et al., 2020). Under these conditions, extracellular public goods are more likely to accumulate locally, thereby protecting otherwise susceptible neighboring bacteria and reducing antibiotic efficacy. Nevertheless, whether the diffusion of public goods observed in well-mixed *in vitro* systems can be directly extrapolated to infected tissues remains uncertain. Unlike laboratory cultures, infected tissues exhibit spatial heterogeneity and localized bacterial communities, which may limit the diffusion of extracellular products and lead to uneven antibiotic exposure (Cremer et al., 2019; Bäcker et al., 2026). In particular, immune-mediated bacterial clearance, inflammatory responses, and nutritional immunity can continuously reshape bacterial cell density and spatial organization, thereby affecting the production, diffusion, and effectiveness of public goods (Leggett et al., 2014). Consequently, cooperative protection is likely to occur only within localized microenvironments rather than uniformly throughout infected tissues.

Beyond serving as a well-established model of CoopAR, *β*-lactamases are clinically important determinants of antibiotic resistance, particularly in ESBL-producing bacteria that pose a significant public health threat (Pitout and Laupland, 2008). Experimental studies have shown that *β*-lactamase-producing bacteria can degrade *β*-lactam antibiotics *in vivo*, thereby protecting otherwise susceptible cells from antibiotic exposure and leading to treatment failure (Brook, 2009). Functional *β*-lactamases produced by commensal *Escherichia coli* have been shown to reduce the clearance of pathogens such as *Listeria monocytogenes* and *Clostridioides difficile* during ampicillin therapy while facilitating their systemic dissemination (Gjonbalaj et al., 2020). In addition, colonization with exogenous extended-spectrum β-lactamase (ESBL)-producing bacteria has been shown to promote the survival of resident microbiota during *β*-lactam treatment and enhanced the emergence of transconjugants upon recolonization (Bakkeren et al., 2021). These findings suggest that cooperative protection not only increases bacterial survival during antibiotic exposure but may also facilitate HGT by prolonging opportunities for plasmid conjugation, thereby accelerating the dissemination of antibiotic resistance determinants within microbial communities. Although these studies provide compelling evidence that CoopAR can occur *in vivo*, they remain few in number, and the extent to which these findings can be generalized to diverse clinical infections remains uncertain.

From a clinical perspective, these experimental insights should be considered for optimized antimicrobial therapy. Strategies that rapidly reduce bacterial burden and minimize opportunities for cooperative interactions may improve treatment outcomes. Optimizing antibiotic pharmacokinetics/pharmacodynamics, particularly by ensuring adequate drug exposure at the infection site and avoiding prolonged periods below the minimum inhibitory concentration (MIC), may limit the ecological conditions that favor cooperative protection. In addition, combination antimicrobial therapy with agents that have complementary mechanisms of action, such as *β*-lactams combined with aminoglycosides or β-lactamase inhibitors, may enhance bacterial killing and reduce the persistence of resistant subpopulations, although direct evidence for suppressing CoopAR remains limited (Tamma et al., 2012; Murugaiyan et al., 2022). Finally, antimicrobial stewardship programs that optimize antibiotic selection, dosing, and treatment duration are expected to reduce the selective pressures that promote the emergence and maintenance of cooperative resistance (Bassetti et al., 2022).

Although increasing evidence demonstrates the existence of CoopAR *in vivo*, its prevalence, mechanisms basis, and clinical significance remain poorly understood. Therapy outcomes are likely influenced by the complex interplay among bacterial densities, prolonged persistence, host immunity, population-level resistance, and extensive microbial interactions (Leggett et al., 2014). Future studies integrating animal models, clinical samples and pharmacokinetic/pharmacodynamic analyses will be essential to determine the conditions under which cooperative resistance contributes to treatment failure and to develop targeted intervention strategies.

## 6 Discussion

Collectively, the available evidence indicates that CoopAR plays a decisive role in bacterial resistance dynamics, challenges the detection and interpretation of resistance phenotypes, and promotes the horizontal spread of resistance determinants. Increasing evidence indicates that bacterial cooperation is not merely a laboratory phenomenon but may substantially affect antibiotic efficacy in polymicrobial communities and in biofilm-associated infections (Bhuiyan, 2025; Nithyanand et al., 2025). Consequently, improving antibiotic efficacy will depend not only on developing more potent antibiotics but also on dismantling CoopAR that allow susceptible bacteria to survive antibiotic treatment (Lissens et al., 2022).

CoopAR is a promising therapeutic target because disrupting bacterial cooperation may improve antibiotic efficacy without necessarily imposing strong selective pressure for resistance. Key strategies include directly targeting public goods, disrupting population-level coordination, exploiting “cheaters,” and leveraging insights from social evolution theory to destabilize cooperative behaviors (Denk-Lobnig and Wood, 2023). For example, quorum-sensing inhibitors, including halogenated furanones and synthetic N-acyl-homoserine lactone analogues, and quorum-quenching enzymes such as acyl-homoserine lactonases and acylases can disrupt bacterial communication by blocking signal perception or degrading extracellular autoinducers (Cui and Kim, 2024). These approaches have been shown to reduce biofilm formation and virulence factor production while enhancing antibiotic efficacy without directly inhibiting bacterial growth. Likewise, *β*-lactamase inhibitors (e.g., clavulanate, avibactam and vaborbactam) block extracellular *β*-lactamase activity, thereby reducing the protection afforded to susceptible neighboring bacteria (Bush and Bradford, 2016). Collectively, these anti-cooperation strategies provide attractive complements to conventional antibiotics by targeting the social interactions that sustain bacterial communities rather than bacterial viability alone.

Because many clinically relevant infections are polymicrobial, effective therapeutic strategies should consider not only individual pathogens but also cooperative interactions within microbial communities. Current evidence suggests that CoopAR may be particularly relevant in chronic pulmonary and wound infections, implant-associated biofilm infections, and gastrointestinal colonization, as these setting are characterized by dense microbial communities, spatial organization, and prolonged antibiotic exposure that can promote cooperative interactions. However, its clinical significance is likely context-dependent, and direct evidence linking CoopAR to treatment outcomes remains limited. Even so, the ecological conditions present in these settings suggest that CoopAR may contribute to persistent infection, treatment failure, and the horizontal transfer of resistance genes.

Beyond promoting resistance, CoopAR mechanisms may also be therapeutically exploited to protect the host microbiota. For example, *β*-lactamase-based approaches, including orally administered enzymes such as SYN-004 and engineered probiotic strains expressing *β*-lactamases, have been used successfully to degrade residual *β*-lactam antibiotics in the gut, thereby preserving the intestinal microbiota and reducing the risk of *Clostridioides difficile* infection and colonization (Connelly et al., 2017; Kokai-Kun et al., 2019; Cubillos-Ruiz et al., 2022).

A growing body of studies has investigated the development of bacterial resistance based on public goods (Dimitriu et al., 2014; Medaney et al., 2016; Frost et al., 2018; Bottery et al., 2021). However, the major challenge is no longer identifying cooperative mechanisms but translating these findings into clinically relevant therapeutic strategies. Most evidence comes from simplified experimental systems that involve single strains, individual public goods, or *in vitro* models, whereas clinical infections are spatially heterogeneous, host-influenced, dynamically exposed to antibiotics, and frequently polymicrobial (Herzberg and van Hasselt, 2025; Hu et al., 2026). Moreover, host immunity and antibiotic pharmacokinetics/pharmacodynamics further shape CoopAR *in vivo*, yet these factors are rarely incorporated into current experimental models. Therefore, there is still a lack of quantitative frameworks to determine which public goods facilitate protection *in vivo*, their effective diffusion range, and the temporal windows when cooperative defenses are most vulnerable. These community-level effects are also largely overlooked by current clinical susceptibility testing.

Together, these findings highlight that CoopAR is an ecological level property of bacterial communities rather than a genetic trait restricted to individual pathogens. This perspective broadens the conventional understanding of antibiotic resistance and underscores the need to of target cooperative interactions alongside resistant bacteria. Integrating microbial ecology, host biology, and clinical pharmacology will be essential for translating mechanistic insights into effective therapeutic strategies and improving the management of polymicrobial infections.

## 7 Future perspectives

By revealing how bacterial cooperation can modulate antibiotic efficacy, CoopAR expands our understanding of resistance beyond classical genetic mechanisms. Despite growing mechanistic insights, major questions remain in understanding its prevalence, ecological determinants, and clinical relevance *in vivo*. Addressing these challenges will require integrated experimental and computational approaches to investigate CoopAR across spatial, cellular, ecological, and clinical scales.

A key priority is to determine how CoopAR operates in clinically relevant infection environments. Unlike *in vitro* planktonic cultures, clinical infections are spatially heterogeneous and shaped by nutrient availability, antibiotic gradients, host responses, and microbial interactions (Bjarnsholt et al., 2022). Combining methods such as spatial metabolomics, advanced imaging, and microfluidic infection models will enable spatial mapping of public goods, signaling molecules, and antibiotics in infected tissues, helping to define the microenvironments in which CoopAR emerges (Zhang et al., 2024; Liu et al., 2025).

Future studies should also resolve the cellular and ecological basis of CoopAR. Single-cell transcriptomics can distinguish producer and beneficiary subpopulations that are obscured in bulk analyses, whereas synthetic microbial communities provide controllable platforms for dissecting interspecies cooperation and metabolic interactions (Cyriaque et al., 2024; Jing et al., 2024; Xiong et al., 2024). Together, these approaches will improve our understanding of how bacterial heterogeneity and community composition influence cooperative resistance.

Finally, integrating multi-omics datasets with systems biology and artificial intelligence (AI) may facilitate prediction of cooperative resistance networks and identification of key public-goods producers, bacterial interactions, and potential therapeutic targets (Tran and Dahlin, 2024; Agarwal and Chumbhale, 2025; Renganathan et al., 2025). Such predictive frameworks could support precision antimicrobial therapy by identifying infection settings where targeting bacterial cooperation is most likely to improve treatment outcomes.

Collectively, these multidisciplinary approaches will provide a more comprehensive understanding of CoopAR and facilitate the translation of ecological insights into clinically relevant strategies to combat antimicrobial resistance.

## References

[ref1] Agarwal A. Chumbhale N. (2025). AI-enhanced multi-omics framework for predicting antibiotic resistance in pediatric cancer patients Avani Agarwal. Res J Cell Sci 1, 1–8. doi: 10.2139/ssrn.5229078

[ref2] Alexandrino M. Costa R. Canário A. V. M. Costa M. C. (2014). Clostridia initiate heavy metal bioremoval in mixed sulfidogenic cultures. Environ. Sci. Technol. 48, 3378–3385. doi: 10.1021/es4052044, 24568215

[ref3] Almatroudi A. (2025). Biofilm resilience: molecular mechanisms driving antibiotic resistance in clinical contexts. Biology 14:165. doi: 10.3390/biology14020165, 40001933 PMC11852148

[ref4] Alves N. J. Turner K. B. Medintz I. L. Walper S. A. (2016). Protecting enzymatic function through directed packaging into bacterial outer membrane vesicles. Sci. Rep. 6, 1–10. doi: 10.1038/srep24866, 27117743 PMC4846811

[ref5] Amanatidou E. Matthews A. C. Kuhlicke U. Neu T. R. McEvoy J. P. Raymond B. (2019). Biofilms facilitate cheating and social exploitation of β-lactam resistance in Escherichia coli. NPJ Biofilms Microbiomes 5:36. doi: 10.1038/s41522-019-0109-2, 31814991 PMC6884583

[ref6] Aslam B. Khurshid M. Arshad M. I. Muzammil S. Rasool M. Yasmeen N. . (2021). Antibiotic resistance: one health one world outlook. Front. Cell. Infect. Microbiol. 11:771510. doi: 10.3389/fcimb.2021.771510, 34900756 PMC8656695

[ref7] Bäcker M. Doekes H. M. Garza D. R. Meijer J. van Vliet S. Allen R. J. (2026). Spatial structure: shaping the ecology and evolution of microbial communities. FEMS Microbiol. Rev. 50:fuaf067. doi: 10.1093/femsre/fuaf067, 41661138 PMC12892361

[ref8] Bakkeren E. Gül E. Huisman J. S. Steiger Y. Rocker A. Hardt W. D. . (2022). Impact of horizontal gene transfer on emergence and stability of cooperative virulence in Salmonella Typhimurium. Nat. Commun. 13:1939. doi: 10.1038/s41467-022-29597-7, 35410999 PMC9001671

[ref9] Bakkeren E. Herter J. A. Huisman J. S. Steiger Y. Gül E. Newson J. P. M. . (2021). Pathogen invasion-dependent tissue reservoirs and plasmid-encoded antibiotic degradation boost plasmid spread in the gut. eLife 10, 1–32. doi: 10.7554/elife.69744, 34872631 PMC8651294

[ref10] Bakkeren E. Huisman J. S. Fattinger S. A. Hausmann A. Furter M. Egli A. . (2019). Salmonella persisters promote the spread of antibiotic resistance plasmids in the gut. Nature 573, 276–280. doi: 10.1038/s41586-019-1521-8, 31485077 PMC6744281

[ref11] Bale B. I. Elebesunu E. E. Manikavasagar P. Agwuna F. O. Ogunkola I. O. Sow A. U. . (2023). Antibiotic resistance in ocular bacterial infections: an integrative review of ophthalmic chloramphenicol. Trop. Med. Health 51:15. doi: 10.1186/s41182-023-00496-x, 36895063 PMC9996861

[ref12] Baltz R. H. (2009). Daptomycin: mechanisms of action and resistance, and biosynthetic engineering. Curr. Opin. Chem. Biol. 13, 144–151. doi: 10.1016/j.cbpa.2009.02.031, 19303806

[ref13] Bassetti S. Tschudin-Sutter S. Egli A. Osthoff M. (2022). Optimizing antibiotic therapies to reduce the risk of bacterial resistance. Eur. J. Intern. Med. 99, 7–12. doi: 10.1016/j.ejim.2022.01.029, 35074246

[ref14] Bernier S. P. Létoffé S. Delepierre M. Ghigo J. (2011). Biogenic ammonia modifies antibiotic resistance at a distance in physically separated bacteria. Mol. Microbiol. 81, 705–716. doi: 10.1111/j.1365-2958.2011.07724.x, 21651627

[ref15] Bhuiyan M. N. I. (2025). Biofilm, resistance, and quorum sensing: the triple threat in bacterial pathogenesis. Microbe 9:100578. doi: 10.1016/j.microb.2025.100578

[ref16] Bielaszewska M. Daniel O. Karch H. Mellmann A. (2020). Dissemination of the blaCTX-M-15 gene among Enterobacteriaceae via outer membrane vesicles. J. Antimicrob. Chemother. 75, 2442–2451. doi: 10.1093/jac/dkaa214, 32562546

[ref17] Bjarnsholt T. Whiteley M. Rumbaugh K. P. Stewart P. S. Jensen P. Frimodt-Møller N. (2022). The importance of understanding the infectious microenvironment. Lancet Infect. Dis. 22, e88–e92. doi: 10.1016/S1473-3099(21)00122-5, 34506737 PMC9190128

[ref18] Blake K. S. Kumar H. Loganathan A. Williford E. E. Diorio-Toth L. Xue Y. P. . (2024). Sequence-structure-function characterization of the emerging tetracycline destructase family of antibiotic resistance enzymes. Commun. Biol. 7:336. doi: 10.1038/s42003-024-06023-w, 38493211 PMC10944477

[ref19] Bottery M. J. Pitchford J. W. Friman V. P. (2021). Ecology and evolution of antimicrobial resistance in bacterial communities. ISME J. 15, 939–948. doi: 10.1038/s41396-020-00832-7, 33219299 PMC8115348

[ref20] Brauner A. Fridman O. Gefen O. Balaban N. Q. (2016). Distinguishing between resistance, tolerance and persistence to antibiotic treatment. Nat. Rev. Microbiol. 14, 320–330. doi: 10.1038/nrmicro.2016.34, 27080241

[ref21] Brepoels P. De Wit G. Lories B. Belpaire T. E. R. Steenackers H. P. (2025). Selective pressures for public antibiotic resistance. Crit. Rev. Microbiol. 51, 417–426. doi: 10.1080/1040841X.2024.2367666, 39158370

[ref22] Brogden K. A. Guthmiller J. M. Taylor C. E. (2005). Human polymicrobial infections. Lancet 365, 253–255. doi: 10.1016/s0140-6736(05)17745-9, 15652608 PMC7119324

[ref23] Brook I. (2004). Β-lactamase-producing Bacteria in mixed infections. Clin. Microbiol. Infect. 10, 777–784. doi: 10.1111/j.1198-743X.2004.00962.x, 15355407

[ref24] Brook I. (2009). The role of beta-lactamase-producing-bacteria in mixed infections. BMC Infect. Dis. 9:202. doi: 10.1186/1471-2334-9-202, 20003454 PMC2804585

[ref25] Buckling A. Brockhurst M. A. (2008). Kin selection and the evolution of virulence. Heredity 100, 484–488. doi: 10.1038/sj.hdy.6801093, 18212805

[ref26] Bush K. (2018). Past and present perspectives on β-lactamases. Antimicrob. Agents Chemother. 62, 1–20. doi: 10.1128/AAC.01076-18, 30061284 PMC6153792

[ref27] Bush K. Bradford P. A. (2016). β-Lactams and β-lactamase inhibitors: an overview. Cold Spring Harb. Perspect. Med. 6:a025247. doi: 10.1101/cshperspect.a025247, 27329032 PMC4968164

[ref28] Castañeda-Barba S. Top E. M. Stalder T. (2024). Plasmids, a molecular cornerstone of antimicrobial resistance in the one health era. Nat. Rev. Microbiol. 22, 18–32. doi: 10.1038/s41579-023-00926-x, 37430173 PMC12440250

[ref29] Charpentier X. Polard P. Claverys J. P. (2012). Induction of competence for genetic transformation by antibiotics: convergent evolution of stress responses in distant bacterial species lacking SOS? Curr. Opin. Microbiol. 15, 570–576. doi: 10.1016/j.mib.2012.08.001, 22910199

[ref30] Che Y. Yang Y. Xu X. Břinda K. Polz M. F. Hanage W. P. . (2021). Conjugative plasmids interact with insertion sequences to shape the horizontal transfer of antimicrobial resistance genes. Proc. Natl. Acad. Sci. 118:e2008731118. doi: 10.1073/pnas.2008731118, 33526659 PMC8017928

[ref31] Choby J. E. Ozturk T. Abbott C. N. Nnabuife C. Colquhoun J. M. Satola S. W. . (2025). Copy number flexibility facilitates heteroresistance to increasing antibiotic pressure and threatens the beta-lactam pipeline. Nat. Commun. 16:5721. doi: 10.1038/s41467-025-60828-9, 40593643 PMC12216759

[ref32] Ciofu O. Tolker-Nielsen T. (2019). Tolerance and resistance of Pseudomonas aeruginosa biofilms to antimicrobial agents-how P. aeruginosa can escape antibiotics. Front. Microbiol. 10:913. doi: 10.3389/fmicb.2019.00913, 31130925 PMC6509751

[ref33] Connelly S. Bristol J. A. Hubert S. Subramanian P. Hasan N. A. Colwell R. R. . (2017). SYN-004 (ribaxamase), an oral beta-lactamase, mitigates antibiotic-mediated dysbiosis in a porcine gut microbiome model. J. Appl. Microbiol. 123, 66–79. doi: 10.1111/jam.13432, 28245091

[ref34] Cremer J. Melbinger A. Wienand K. Henriquez T. Jung H. Frey E. (2019). Cooperation in microbial populations: theory and experimental model systems. J. Mol. Biol. 431, 4599–4644. doi: 10.1016/j.jmb.2019.09.023, 31634468

[ref35] Cubillos-Ruiz A. Alcantar M. A. Donghia N. M. Cárdenas P. Avila-Pacheco J. Collins J. J. (2022). An engineered live biotherapeutic for the prevention of antibiotic-induced dysbiosis. Nat. Biomed. Eng. 6, 910–921. doi: 10.1038/s41551-022-00871-9 35411114

[ref36] Cui S. Kim E. (2024). Quorum sensing and antibiotic resistance in polymicrobial infections. Commun. Integr. Biol. 17:2415598. doi: 10.1080/19420889.2024.2415598, 39430726 PMC11487952

[ref37] Cyriaque V. Ibarra-Chávez R. Kuchina A. Seelig G. Nesme J. Madsen J. S. (2024). Single-cell RNA sequencing reveals plasmid constrains bacterial population heterogeneity and identifies a non-conjugating subpopulation. Nat. Commun. 15:5853. doi: 10.1038/s41467-024-49793-x, 38997267 PMC11245611

[ref38] Denk-Lobnig M. Wood K. B. (2023). Antibiotic resistance in bacterial communities. Curr. Opin. Microbiol. 74:102306. doi: 10.1016/j.mib.2023.102306, 37054512 PMC10527032

[ref39] Denk-Lobnig M. K. Wood K. B. (2025). Spatial population dynamics of bacterial colonies with social antibiotic resistance. Proc. Natl. Acad. Sci. USA 122:e2417065122. doi: 10.1073/pnas.2417065122, 39937854 PMC11848446

[ref40] Devos S. Van Oudenhove L. Stremersch S. Van Putte W. De Rycke R. Van Driessche G. . (2015). The effect of imipenem and diffusible signaling factors on the secretion of outer membrane vesicles and associated Ax21 proteins in Stenotrophomonas maltophilia. Front. Microbiol. 6, 1–9. doi: 10.3389/fmicb.2015.00298, 25926824 PMC4396451

[ref41] Dewar A. E. Thomas J. L. Scott T. W. Wild G. Griffin A. S. West S. A. . (2021). Plasmids do not consistently stabilize cooperation across bacteria but may promote broad pathogen host-range. Nat. Ecol. Evol. 5, 1624–1636. doi: 10.1038/s41559-021-01573-2, 34750532 PMC7612097

[ref42] Diggle S. P. Griffin A. S. Campbell G. S. West S. A. (2007). Cooperation and conflict in quorum-sensing bacterial populations. Nature 450, 411–414. doi: 10.1038/nature06279 18004383

[ref43] Dimitriu T. Lotton C. Beńard-Capelle J. Misevic D. Brown S. P. Lindner A. B. . (2014). Genetic information transfer promotes cooperation in bacteria. Proc. Natl. Acad. Sci. USA 111, 11103–11108. doi: 10.1073/pnas.1406840111, 25024219 PMC4121819

[ref44] Dimitriu T. Medaney F. Amanatidou E. Forsyth J. Ellis R. J. Raymond B. (2019). Negative frequency dependent selection on plasmid carriage and low fitness costs maintain extended spectrum β-lactamases in Escherichia coli. Sci. Rep. 9, 1–7. doi: 10.1038/s41598-019-53575-7, 31748602 PMC6868128

[ref45] Domingues I. L. Gama J. A. Carvalho L. M. Dionisio F. (2017). Social behaviour involving drug resistance: the role of initial density, initial frequency and population structure in shaping the effect of antibiotic resistance as a public good. Heredity 119, 295–301. doi: 10.1038/hdy.2017.33, 28635967 PMC5637362

[ref46] Domingues S. Nielsen K. M. (2017). Membrane vesicles and horizontal gene transfer in prokaryotes. Curr. Opin. Microbiol. 38, 16–21. doi: 10.1016/j.mib.2017.03.012, 28441577

[ref47] Donaldson G. P. Lee S. M. Mazmanian S. K. (2016). Gut biogeography of the bacterial microbiota. Nat. Rev. Microbiol. 14, 20–32. doi: 10.1038/nrmicro3552, 26499895 PMC4837114

[ref48] Doublet B. Boyd D. Douard G. Praud K. Cloeckaert A. Mulvey M. R. (2012). Complete nucleotide sequence of the multidrug resistance IncA/C plasmid pR55 from Klebsiella pneumoniae isolated in 1969. J. Antimicrob. Chemother. 67, 2354–2360. doi: 10.1093/jac/dks251, 22773739

[ref49] Elshobary M. E. Badawy N. K. Ashraf Y. Zatioun A. A. Masriya H. H. Ammar M. M. . (2025). Combating antibiotic resistance: mechanisms, multidrug-resistant pathogens, and novel therapeutic approaches: an updated review. Pharmaceuticals 18:402. doi: 10.3390/ph18030402, 40143178 PMC11944582

[ref50] Frost I. Smith W. P. J. Mitri S. Millan A. S. Davit Y. Osborne J. M. . (2018). Cooperation, competition and antibiotic resistance in bacterial colonies. ISME J. 12, 1582–1593. doi: 10.1038/s41396-018-0090-4, 29563570 PMC5955900

[ref51] Geyrhofer L. Ruelens P. Farr A. D. Pesce D. de Visser J. A. G. M. Brenner N. (2023). Minimal surviving inoculum in collective antibiotic resistance. MBio 14:e02456-22. doi: 10.1128/mbio.02456-22, 37022160 PMC10128016

[ref52] Gjonbalaj M. Keith J. W. Do M. H. Hohl T. M. Pamer E. G. Becattini S. (2020). Antibiotic degradation by commensal microbes shields pathogens. Infect. Immun. 88, 10–1128. doi: 10.1128/IAI.00012-20, 31964746 PMC7093146

[ref53] González L. J. Bahr G. Nakashige T. G. Nolan E. M. Bonomo R. A. Vila A. J. (2016). Membrane anchoring stabilizes and favors secretion of New Delhi metallo-β-lactamase. Nat. Chem. Biol. 12, 516–522. doi: 10.1038/nchembio.2083, 27182662 PMC4912412

[ref54] Griffin A. S. Leeks A. (2025). Exploiting social traits for clinical applications in bacteria and viruses. NPJ Antimicrob. Resist 3:20. doi: 10.1038/s44259-025-00091-6 40155763 PMC11953253

[ref9001] Groenhagen U. Baumgartner R. Bailly A. Gardiner A. Eberl L. Schulz S. . (2013). Production of Bioactive Volatiles by Different Burkholderia ambifaria Strains. J. Chem. Ecol. 39, 892–906. doi: 10.1007/s10886-013-0315-y, 23832658

[ref55] Gunn J. S. Bakaletz L. O. Wozniak D. J. (2016). What’s on the outside matters: the role of the extracellular polymeric substance of gram-negative biofilms in evading host immunity and as a target for therapeutic intervention. J. Biol. Chem. 291, 12538–12546. doi: 10.1074/jbc.R115.707547, 27129225 PMC4933457

[ref56] Hamilton W. D. (1964). The genetical evolution of social behaviour. II. J. Theor. Biol. 7, 17–52. doi: 10.1016/0022-5193(64)90039-6, 5875340

[ref57] Hernández-Navarro L. Asker M. Rucklidge A. M. Mobilia M. (2023). Coupled environmental and demographic fluctuations shape the evolution of cooperative antimicrobial resistance. J. R. Soc. Interface 20:20230393. doi: 10.1098/rsif.2023.0393, 37907094 PMC10618063

[ref58] Hernández-Navarro L. Distefano K. Täuber U. C. Mobilia M. (2026). Slow spatial migration can help eradicate cooperative antimicrobial resistance in time-varying environments. PLoS Comput. Biol. 22:e1013997. doi: 10.1371/journal.pcbi.1013997, 41838786 PMC13132466

[ref59] Herzberg C. van Hasselt J. G. C. (2025). Pharmacodynamics of interspecies interactions in polymicrobial infections. NPJ Biofilms Microbiomes 11:20. doi: 10.1038/s41522-024-00621-6, 39837846 PMC11751299

[ref60] Hu Z. Wu Y. Freire T. Gjini E. Wood K. (2026). Linking spatial drug heterogeneity to microbial growth dynamics in theory and experiment. PLoS Comput. Biol. 22:e1013896. doi: 10.1371/journal.pcbi.1013896, 41557766 PMC12863682

[ref61] Huemer M. Mairpady Shambat S. Brugger S. D. Zinkernagel A. S. (2020). Antibiotic resistance and persistence—implications for human health and treatment perspectives. EMBO Rep. 21, 1–24. doi: 10.15252/embr.202051034, 33400359 PMC7726816

[ref62] Jing J. Garbeva P. Raaijmakers J. M. Medema M. H. (2024). Strategies for tailoring functional microbial synthetic communities. ISME J. 18:wrae049. doi: 10.1093/ismejo/wrae049, 38537571 PMC11008692

[ref63] Khan A. Davlieva M. Panesso D. Rincon S. Miller W. R. Diaz L. . (2019). Antimicrobial sensing coupled with cell membrane remodeling mediates antibiotic resistance and virulence in Enterococcus faecalis. Proc. Natl. Acad. Sci. USA 116, 26925–26932. doi: 10.1073/pnas.1916037116, 31818937 PMC6936494

[ref64] Kim K. Lee S. Ryu C.-M. (2013). Interspecific bacterial sensing through airborne signals modulates locomotion and drug resistance. Nat. Commun. 4, 1–12. doi: 10.1038/ncomms2789, 23651997

[ref65] Kim S. W. Park S. B. Im S. P. Lee J. S. Jung J. W. Gong T. W. . (2018). Outer membrane vesicles from β-lactam-resistant Escherichia coli enable the survival of β-lactam-susceptible E. coli in the presence of β-lactam antibiotics. Sci. Rep. 8, 5402–5413. doi: 10.1038/s41598-018-23656-0, 29599474 PMC5876404

[ref66] Kim S. W. Seo J. S. Park S. B. Lee A. R. Lee J. S. Jung J. W. . (2020). Significant increase in the secretion of extracellular vesicles and antibiotics resistance from methicillin-resistant Staphylococcus aureus induced by ampicillin stress. Sci. Rep. 10, 21066–21014. doi: 10.1038/s41598-020-78121-8, 33273518 PMC7713300

[ref67] Kokai-Kun J. F. Roberts T. Coughlin O. Le C. Whalen H. Stevenson R. . (2019). Use of ribaxamase (SYN-004), a β-lactamase, to prevent Clostridium difficile infection in β-lactam-treated patients: a double-blind, phase 2b, randomised placebo-controlled trial. Lancet Infect. Dis. 19, 487–496. doi: 10.1016/S1473-3099(18)30731-X, 30885591

[ref68] Kolling G. L. Matthews K. R. (1999). Export of virulence genes and Shiga toxin by membrane vesicles of Escherichia coli O157:H7. Appl. Environ. Microbiol. 65, 1843–1848. doi: 10.1128/aem.65.5.1843-1848.1999, 10223967 PMC91264

[ref69] Lee I. P. A. Eldakar O. T. Gogarten J. P. Andam C. P. (2021). Bacterial cooperation through horizontal gene transfer. Trends Ecol. Evol. 37, 223–232. doi: 10.1016/j.tree.2021.11.006, 34815098

[ref70] Leggett H. C. Brown S. P. Reece S. E. (2014). War and peace: social interactions in infections. Philos Trans R Soc Lond B Biol Sci 369:20130365. doi: 10.1098/rstb.2013.0365, 24686936 PMC3982666

[ref71] León-Sampedro R. DelaFuente J. Díaz-Agero C. Crellen T. Musicha P. Rodríguez-Beltrán J. . (2021). Pervasive transmission of a carbapenem resistance plasmid in the gut microbiota of hospitalized patients. Nat. Microbiol. 6, 606–616. doi: 10.1038/s41564-021-00879-y, 33782584 PMC7610705

[ref72] Lerminiaux N. A. Cameron A. D. S. (2019). Horizontal transfer of antibiotic resistance genes in clinical environments. Can. J. Microbiol. 65, 34–44. doi: 10.1139/cjm-2018-0275, 30248271

[ref73] Létoffé S. Audrain B. Bernier S. P. Delepierre M. Ghigo J. M. (2014). Aerial exposure to the bacterial volatile compound trimethylamine modifies antibiotic resistance of physically separated bacteria by raising culture medium pH. MBio 5:e00944-13. doi: 10.1128/mBio.00944-13, 24399857 PMC3884056

[ref74] Lim S. Yoon H. (2015). Roles of outer membrane vesicles (OMVs) in bacterial virulence. J. Bacteriol. Virol. 45, 1–10. doi: 10.4167/jbv.2015.45.1.1

[ref75] Lissens M. Joos M. Lories B. Steenackers H. P. (2022). Evolution-proof inhibitors of public good cooperation: a screening strategy inspired by social evolution theory. FEMS Microbiol. Rev. 46, 1–18. doi: 10.1093/femsre/fuac019, 35675280 PMC9616471

[ref76] Liu T. Fu H. Shi C. Jiang Y. Yu C. Guo C. . (2025). Mass spectrometry imaging: unveiling new horizons in antibiotic research. Crit. Rev. Anal. Chem., 1–16. doi: 10.1080/10408347.2025.2599309, 41354940

[ref77] Liu H. Y. Prentice E. L. Webber M. A. (2024). Mechanisms of antimicrobial resistance in biofilms. NPJ Antimicrob Resist 2:27. doi: 10.1038/s44259-024-00046-3, 39364333 PMC11445061

[ref78] Livermore D. M. (1995). beta-lactamases in laboratory and clinical resistance. Clin. Microbiol. Rev. 8, 557–584. doi: 10.1128/CMR.8.4.557, 8665470 PMC172876

[ref79] Ma H. R. Xu H. Z. Kim K. Anderson D. J. You L. (2024). Private benefit of β-lactamase dictates selection dynamics of combination antibiotic treatment. Nat. Commun. 15:8337. doi: 10.1038/s41467-024-52711-w, 39333122 PMC11436977

[ref80] Madsen J. S. Sørensen S. J. Burmølle M. (2018). Bacterial social interactions and the emergence of community-intrinsic properties. Curr. Opin. Microbiol. 42, 104–109. doi: 10.1016/j.mib.2017.11.018, 29197823

[ref81] Magnet S. Blanchard J. S. (2005). Molecular insights into aminoglycoside action and resistance. Chem. Rev. 105, 477–498. doi: 10.1021/cr0301088, 15700953

[ref82] Mc Ginty S. É. Lehmann L. Brown S. P. Rankin D. J. (2013). The interplay between relatedness and horizontal gene transfer drives the evolution of plasmid-carried public goods. Proc. R. Soc. B Biol. Sci. 280:20130400. doi: 10.1098/rspb.2013.0400, 23760639 PMC3652439

[ref83] Medaney F. Dimitriu T. Ellis R. J. Raymond B. (2016). Live to cheat another day: bacterial dormancy facilitates the social exploitation of β-lactamases. ISME J. 10, 778–787. doi: 10.1038/ismej.2015.154, 26505830 PMC4817691

[ref84] Millan A. S. Escudero J. A. Gifford D. R. Mazel D. MacLean R. C. (2016). Multicopy plasmids potentiate the evolution of antibiotic resistance in bacteria. Nat. Ecol. Evol. 1:10. doi: 10.1038/s41559-016-0010, 28812563

[ref85] Moffa M. Brook I. (2015). “Tetracyclines, Glycylcyclines, and chloramphenicol,” in Mandell, Douglas, and Bennett’s Principles and Practice of Infectious Diseases, (Amsterdam: Elsevier), 322–338.e6.

[ref86] Molina-Santiago C. Daddaoua A. Fillet S. Duque E. Ramos J. L. (2014). Interspecies signalling: Pseudomonas putida efflux pump TtgGHI is activated by indole to increase antibiotic resistance. Environ. Microbiol. 16, 1267–1281. doi: 10.1111/1462-2920.12368, 24373097

[ref87] Murray C. J. Ikuta K. S. Sharara F. Swetschinski L. Robles Aguilar G. Gray A. . (2022). Global burden of bacterial antimicrobial resistance in 2019: a systematic analysis. Lancet 399, 629–655. doi: 10.1016/s0140-6736(21)02724-0, 35065702 PMC8841637

[ref88] Murray I. A. Shaw W. V. (1997). O-acetyltransferases for chloramphenicol and other natural products. Antimicrob. Agents Chemother. 41, 1–6. doi: 10.1128/aac.41.1.1, 8980745 PMC163650

[ref89] Murugaiyan J. Anand Kumar P. Rao G. S. Iskandar K. Hawser S. Hays J. P. . (2022). Progress in alternative strategies to combat antimicrobial resistance: focus on antibiotics. Antibiotics 11:200. doi: 10.3390/antibiotics11020200, 35203804 PMC8868457

[ref90] Naghavi M. Emil Vollset S. Ikuta K. S. Swetschinski L. R. Gray A. P. Wool E. E. . (2024). Global burden of bacterial antimicrobial resistance 1990–2021: a systematic analysis with forecasts to 2050. Lancet 404, 1199–1226. doi: 10.1016/S0140-6736(24)01867-1 39299261 PMC11718157

[ref91] Nicoloff H. H. Andersson D. I. (2016). Indirect resistance to several classes of antibiotics in cocultures with resistant bacteria expressing antibiotic-modifying or -degrading enzymes. J. Antimicrob. Chemother. 71, 100–110. doi: 10.1093/jac/dkv312 26467993

[ref92] Nicoloff H. Hjort K. Andersson D. I. Wang H. (2024). Three concurrent mechanisms generate gene copy number variation and transient antibiotic heteroresistance. Nat. Commun. 15:3981. doi: 10.1038/s41467-024-48233-0, 38730266 PMC11087502

[ref93] Nithyanand P. Boya B. R. Lee J. Lee J. (2025). Polymicrobial biofilms: interkingdom interactions, resistance and therapeutic strategies. Microb. Biotechnol. 18:18. doi: 10.1111/1751-7915.70218, 40847578 PMC12373980

[ref94] Niu H. Gu J. Zhang Y. (2024). Bacterial persisters: molecular mechanisms and therapeutic development. Signal Transduct. Target. Ther. 9:174. doi: 10.1038/s41392-024-01866-5, 39013893 PMC11252167

[ref95] Nogueira T. Rankin D. J. Touchon M. Taddei F. Brown S. P. Rocha E. P. C. (2009). Horizontal gene transfer of the Secretome drives the evolution of bacterial cooperation and virulence. Curr. Biol. 19, 1683–1691. doi: 10.1016/j.cub.2009.08.056, 19800234 PMC2773837

[ref96] Pader V. Hakim S. Painter K. L. Wigneshweraraj S. Clarke T. B. Edwards A. M. (2016). Staphylococcus aureus inactivates daptomycin by releasing membrane phospholipids. Nat. Microbiol. 2:16194. doi: 10.1038/nmicrobiol.2016.194, 27775684

[ref97] Perlin M. H. Clark D. R. McKenzie C. Patel H. Jackson N. Kormanik C. . (2009). Protection of Salmonella by ampicillin-resistant Escherichia coli in the presence of otherwise lethal drug concentrations. Proc. R. Soc. B Biol. Sci. 276, 3759–3768. doi: 10.1098/rspb.2009.0997, 19656787 PMC2817278

[ref98] Perry E. K. Meirelles L. A. Newman D. K. (2022). From the soil to the clinic: the impact of microbial secondary metabolites on antibiotic tolerance and resistance. Nat. Rev. Microbiol. 20, 129–142. doi: 10.1038/s41579-021-00620-w, 34531577 PMC8857043

[ref99] Pitout J. D. Laupland K. B. (2008). Extended-spectrum β-lactamase-producing Enterobacteriaceae: an emerging public-health concern. Lancet Infect. Dis. 8, 159–166. doi: 10.1016/S1473-3099(08)70041-0, 18291338

[ref100] Prunotto A. Bahr G. González L. J. Vila A. J. Dal Peraro M. (2020). Molecular bases of the membrane association mechanism potentiating antibiotic resistance by New Delhi Metallo-β-lactamase 1. ACS Infect. Dis. 6, 2719–2731. doi: 10.1021/acsinfecdis.0c00341, 32865963 PMC9062785

[ref101] Que Y. A. Hazan R. Strobel B. Maura D. He J. Kesarwani M. . (2013). A quorum sensing small volatile molecule promotes antibiotic tolerance in bacteria. PLoS One 8, 1–9. doi: 10.1371/journal.pone.0080140, 24367477 PMC3868577

[ref102] Rahal J. J. Jr. Simberkoff M. S. (1979). Bactericidal and bacteriostatic action of chloramphenicol against meningeal pathogens. Antimicrob. Agents Chemother. 16, 13–18. doi: 10.1128/AAC.16.1.13, 38742 PMC352780

[ref103] Renganathan P. Gaysina L. A. García Gutiérrez C. Rueda Puente E. O. Sainz-Hernández J. C. (2025). Harnessing engineered microbial consortia for xenobiotic bioremediation: integrating multi-omics and AI for next-generation wastewater treatment. J. Xenobiot. 15:133. doi: 10.3390/jox15040133, 40863340 PMC12387628

[ref104] Roch M. Sierra R. Andrey D. O. (2023). Antibiotic heteroresistance in ESKAPE pathogens, from bench to bedside. Clin. Microbiol. Infect. 29, 320–325. doi: 10.1016/j.cmi.2022.10.018, 36270588

[ref105] Rodríguez-Beltrán J. DelaFuente J. León-Sampedro R. MacLean R. C. San Millán Á. (2021). Beyond horizontal gene transfer: the role of plasmids in bacterial evolution. Nat. Rev. Microbiol. 19, 347–359. doi: 10.1038/s41579-020-00497-1, 33469168

[ref106] Rozwandowicz M. Brouwer M. S. M. Fischer J. Wagenaar J. A. Gonzalez-Zorn B. Guerra B. . (2018). Plasmids carrying antimicrobial resistance genes in Enterobacteriaceae. J. Antimicrob. Chemother. 73, 1121–1137. doi: 10.1093/jac/dkx488, 29370371

[ref107] Rumbo C. Fernández-Moreira E. Merino M. Poza M. Mendez J. A. Soares N. C. . (2011). Horizontal transfer of the OXA-24 carbapenemase gene via outer membrane vesicles: a new mechanism of dissemination of carbapenem resistance genes in Acinetobacter baumannii. Antimicrob. Agents Chemother. 55, 3084–3090. doi: 10.1128/AAC.00929-10, 21518847 PMC3122458

[ref108] Sanz-García F. Gil-Gil T. Laborda P. Blanco P. Ochoa-Sánchez L.-E. Baquero F. . (2023). Translating eco-evolutionary biology into therapy to tackle antibiotic resistance. Nat. Rev. Microbiol. 21, 671–685. doi: 10.1038/s41579-023-00902-5, 37208461

[ref109] Schaar V. Nordström T. Mörgelin M. Riesbeck K. (2011). Moraxella catarrhalis outer membrane vesicles carry β-lactamase and promote survival of Streptococcus pneumoniae and Haemophilus influenzae by inactivating amoxicillin. Antimicrob. Agents Chemother. 55, 3845–3853. doi: 10.1128/AAC.01772-10, 21576428 PMC3147650

[ref110] Schaar V. Paulsson M. Mörgelin M. Riesbeck K. (2013). Outer membrane vesicles shield Moraxella catarrhalis β-lactamase from neutralization by serum IgG. J. Antimicrob. Chemother. 68, 593–600. doi: 10.1093/jac/dks444, 23184710

[ref111] Schaar V. Uddbäck I. Nordström T. Riesbeck K. (2014). Group a streptococci are protected from amoxicillin-mediated killing by vesicles containing β-lactamase derived from Haemophilus influenzae. J. Antimicrob. Chemother. 69, 117–120. doi: 10.1093/jac/dkt307, 23912886

[ref112] Schwechheimer C. Kuehn M. J. (2015). Outer-membrane vesicles from gram-negative bacteria: biogenesis and functions. Nat. Rev. Microbiol. 13, 605–619. doi: 10.1038/nrmicro3525, 26373371 PMC5308417

[ref113] Shatalin K. Shatalina E. Mironov A. Nudler E. (2011). H2S: a universal defense against antibiotics in bacteria. Science 334, 986–990. doi: 10.1126/science.1209855, 22096201

[ref114] Shaw W. V. (1983). Chloramphenicol acetyltransferase: enzymology and molecular biolog. Crit. Rev. Biochem. Mol. Biol. 14, 1–46. doi: 10.3109/10409238309102789, 6340955

[ref115] Smith P. Schuster M. (2019). Public goods and cheating in microbes. Curr. Biol. 29, R442–R447. doi: 10.1016/j.cub.2019.03.001, 31163154

[ref116] Soler N. Forterre P. (2020). Vesiduction: the fourth way of HGT. Environ. Microbiol. 22, 2457–2460. doi: 10.1111/1462-2920.15056, 32363801

[ref117] Sørensen S. J. Bailey M. Hansen L. H. Kroer N. Wuertz S. (2005). Studying plasmid horizontal transfer in situ: a critical review. Nat. Rev. Microbiol. 3, 700–710. doi: 10.1038/nrmicro1232, 16138098

[ref118] Sorg R. A. Lin L. van Doorn G. S. Sorg M. Olson J. Nizet V. . (2016). Collective resistance in microbial communities by intracellular antibiotic deactivation. PLoS Biol. 14, e2000631–e2000619. doi: 10.1371/journal.pbio.2000631, 28027306 PMC5189934

[ref119] Stentz R. Horn N. Cross K. Salt L. Brearley C. Livermore D. M. . (2015). Cephalosporinases associated with outer membrane vesicles released by Bacteroides spp. protect gut pathogens and commensals against β-lactam antibiotics. J. Antimicrob. Chemother. 70, 701–709. doi: 10.1093/jac/dku466, 25433011 PMC4319488

[ref120] Tamma P. D. Cosgrove S. E. Maragakis L. L. (2012). Combination therapy for treatment of infections with gram-negative bacteria. Clin. Microbiol. Rev. 25, 450–470. doi: 10.1128/CMR.05041-11, 22763634 PMC3416487

[ref121] Tooke C. L. Hinchliffe P. Bragginton E. C. Colenso C. K. Hirvonen V. H. A. Takebayashi Y. . (2019). β-Lactamases and β-lactamase inhibitors in the 21st century. J. Mol. Biol. 431, 3472–3500. doi: 10.1016/j.jmb.2019.04.002, 30959050 PMC6723624

[ref122] Toyofuku M. Nomura N. Eberl L. (2019). Types and origins of bacterial membrane vesicles. Nat. Rev. Microbiol. 17, 13–24. doi: 10.1038/s41579-018-0112-2, 30397270

[ref123] Tran D. T. Dahlin A. (2024). “Multi-omics approaches to resolve antimicrobial resistance,” in Antimicrobial Resistance: Factors to Findings, eds. Soni V. Akhade A. S. (Cham: Springer International Publishing), 275–294. doi: 10.1007/978-3-031-65986-7_8

[ref124] Vega N. M. Gore J. (2014). Collective antibiotic resistance: mechanisms and implications. Curr. Opin. Microbiol. 21, 28–34. doi: 10.1016/j.mib.2014.09.003, 25271119 PMC4367450

[ref125] Villa L. Capone A. Fortini D. Dolejska M. Rodríguez I. Taglietti F. . (2013). Reversion to susceptibility of a carbapenem-resistant clinical isolate of Klebsiella pneumoniae producing KPC-3. J. Antimicrob. Chemother. 68, 2482–2486. doi: 10.1093/jac/dkt235, 23800906

[ref126] Villa L. Poirel L. Nordmann P. Carta C. Carattoli A. (2012). Complete sequencing of an IncH plasmid carrying the Bla NDM-1, Bla CTX-M-15 and qnrB1 genes. J. Antimicrob. Chemother. 67, 1645–1650. doi: 10.1093/jac/dks114, 22511638

[ref127] Von Wintersdorff C. J. H. Penders J. Van Niekerk J. M. Mills N. D. Majumder S. Van Alphen L. B. . (2016). Dissemination of antimicrobial resistance in microbial ecosystems through horizontal gene transfer. Front. Microbiol. 7:173. doi: 10.3389/fmicb.2016.00173, 26925045 PMC4759269

[ref128] Wang Q. Wei S. Madsen J. S. (2025). Cooperative resistance varies among β-lactamases in E. coli, with some enabling cross-protection and sustained extracellular activity. Commun. Biol. 8:968. doi: 10.1038/s42003-025-08392-2, 40595357 PMC12217783

[ref129] Wang Q. Wei S. Silva A. F. Madsen J. S. (2023). Cooperative antibiotic resistance facilitates horizontal gene transfer. ISME J. 17, 846–854. doi: 10.1038/s41396-023-01393-1, 36949153 PMC10203111

[ref130] Weisskopf L. Schulz S. Garbeva P. (2021). Microbial volatile organic compounds in intra-kingdom and inter-kingdom interactions. Nat. Rev. Microbiol. 19, 391–404. doi: 10.1038/s41579-020-00508-1, 33526910

[ref131] Windels E. M. Michiels J. E. Fauvart M. Wenseleers T. Van den Bergh B. Michiels J. (2019). Bacterial persistence promotes the evolution of antibiotic resistance by increasing survival and mutation rates. ISME J. 13, 1239–1251. doi: 10.1038/s41396-019-0344-9, 30647458 PMC6474225

[ref132] Xiong X. Othmer H. G. Harcombe W. R. (2024). Emergent antibiotic persistence in a spatially structured synthetic microbial mutualism. ISME J. 18:wrae075. doi: 10.1093/ismejo/wrae075, 38691424 PMC11104777

[ref133] Yáñez-Mó M. Siljander P. R. M. Andreu Z. Zavec A. B. Borràs F. E. Buzas E. I. . (2015). Biological properties of extracellular vesicles and their physiological functions. J. Extracell. Vesicles 4:27066. doi: 10.3402/jev.v4.27066, 25979354 PMC4433489

[ref134] Yaron S. Kolling G. L. Simon L. Matthews K. R. (2000). Vesicle-mediated transfer of virulence genes from Escherichia coli O157:H7 to other enteric bacteria. Appl. Environ. Microbiol. 66, 4414–4420. doi: 10.1128/AEM.66.10.4414-4420.2000, 11010892 PMC92318

[ref135] Yin J. Wang G. Cheng D. Fu J. Qiu J. Yu Z. (2019). Inactivation of polymyxin by hydrolytic mechanism. Antimicrob. Agents Chemother. 63, 1–10. doi: 10.1128/AAC.02378-18, 30936102 PMC6535509

[ref136] Yu J. S. L. Correia-Melo C. Zorrilla F. Herrera-Dominguez L. Wu M. Y. Hartl J. . (2022). Microbial communities form rich extracellular metabolomes that foster metabolic interactions and promote drug tolerance. Nat. Microbiol. 7, 542–555. doi: 10.1038/s41564-022-01072-5, 35314781 PMC8975748

[ref137] Yurtsev E. A. Chao H. X. Datta M. S. Artemova T. Gore J. (2013). Bacterial cheating drives the population dynamics of cooperative antibiotic resistance plasmids. Mol. Syst. Biol. 9, 683–687. doi: 10.1038/msb.2013.39, 23917989 PMC3779801

[ref138] Yurtsev E. A. Conwill A. Gore J. (2016). Oscillatory dynamics in a bacterial cross-protection mutualism. Proc. Natl. Acad. Sci. USA 113, 6236–6241. doi: 10.1073/pnas.1523317113, 27194723 PMC4896713

[ref139] Zhang Y. Cai Y. Zhang B. Zhang Y. H. P. J. (2024). Spatially structured exchange of metabolites enhances bacterial survival and resilience in biofilms. Nat. Commun. 15:7575. doi: 10.1038/s41467-024-51940-3, 39217184 PMC11366000

[ref140] Zhang L. Li S. Liu Z. Zhang R. Yang T. Liu D. . (2026). Microbial interactions in facilitating antibiotic activity and resistance evolution. Appl. Environ. Microbiol. 92:e0193125. doi: 10.1128/aem.01931-25, 41489360 PMC12838386

[ref141] Zhao X. Ruelens P. Farr A. D. de Visser J. A. G. M. Baraban L. (2023). Population dynamics of cross-protection against β-lactam antibiotics in droplet microreactors. Front. Microbiol. 14:1294790. doi: 10.3389/fmicb.2023.1294790, 38192289 PMC10773670

[ref142] Zhu S. Hong J. Wang T. (2024). Horizontal gene transfer is predicted to overcome the diversity limit of competing microbial species. Nat. Commun. 15:800. doi: 10.1038/s41467-024-45154-w, 38280843 PMC10821886

